# Ophthalmic viscosurgical device interaction with two hydrophobic acrylic intraocular lenses of different equilibrium water content

**DOI:** 10.1038/s41598-022-18813-5

**Published:** 2022-08-26

**Authors:** Gerd U. Auffarth, Sonja K. Schickhardt, Hui Fang, Qiang Wang, Ramin Khoramnia, Timur M. Yildirim

**Affiliations:** 1 The David J Apple International Laboratory for Ocular Pathology, Department of Ophthalmology, University of Heidelberg, 69120 Heidelberg, Germany; 2 Department of Ophthalmology, Third Affiliated Hospital, Wenzhou Medical University, Rui’an, 325200 Zhejiang China

**Keywords:** Medical research, Chemistry

## Abstract

Ophthalmic viscosurgical device (OVD) is used during intraocular surgery to protect ocular tissue. It requires complete removal from the eye by the end of surgery to avoid postoperative complications. This study compares the interaction of a cohesive OVD with two different intraocular lenses (IOLs) of different equilibrium water content. In this laboratory study on porcine cadaver eyes, the capsular bags and anterior chambers of each eye were filled with fluorescein-stained OVD. Following implantation of 10 IOLs each of Clareon CNA0T0 and AcrySof SN60WF (Alcon Laboratory, Fort Worth, USA) IOLs, the OVD was removed using the irrigation/aspiration mode. The OVD removal was timed and differences between the both IOL groups were compared. OVD removal time ranged from 18 to 40 s (mean ± SD, 26.4 ± 6.8 s) and from 16 to 39 s (mean ± SD, 23.6 ± 6.6 s) for eyes implanted with a CNA0T0 and a SN60WF IOL, respectively, without a statistically significant difference between the groups, P > 0.05. Cohesive OVD removal times were similar between the CNA0T0 and SN60WF groups. Surgeons should experience no differences regarding the interaction between cohesive OVDs and IOLs made from the new Clareon material compared to the established AcrySof material.

## Introduction

Cataract surgery is facilitated by ophthalmic viscosurgical devices (OVDs)^1–3^. After the intraocular lens (IOL) is implanted, the OVD must be removed completely from the eye as any remaining OVD can lead to postoperative complications, such as increased intraocular pressure (IOP), endothelial cell loss or refractive shifts^4^. The material of the IOL implant interacts with the OVD which can affect its removal behaviour^1^. Hydrophobic IOLs usually incorporate a minimal amount of water. The lowest water content is found in lenses made from polymethyl methacrylate (PMMA). A well-established IOL made from a hydrophobic acrylic copolymer is the AcrySof (Alcon Laboratories, Inc., Fort Worth, TX, USA) with an equilibrium water content (EWC) of around 0.5%. Hydrophobic acrylic IOLs have shown to develop an undesired late postoperative material change which is the formation of fluid-filled microvacoules within the IOL polymer. For more than 20 years, IOL manufacturers have been trying to eliminate the occurance of theses so called glistenings and subsurface nanoglistenings (SSNG) in their IOL materials. With the Clareon (Alcon Laboratories, Inc., Fort Worth, TX, USA) the Alcon company created a new material formulation that aims to eliminate the complication of glistening and SSNG formation. Clareon is made of a hydrophobic acrylic copolymer with a EWC of 1.5% at 35 °C and a refractive index of 1.55. The Clareon CNA0T0 has an overall length of 13.0 mm and a full 6.0 mm functional biconvex aspheric optic. The design of the lens was based on the mechanically stable platform of the single-piece AcrySof SN60WF IOL (Alcon)^18^.

The time required to remove an OVD correlates with both, composition of the IOL material and type of OVD, which is usually catigorized in either dispersive or cohesive^1^. OVD removal times for different lens materials, such silicone, PMMA and other acrylic materials have been evaluated in previous studies^1,2^. PMMA was found to yield a shorter removal time for cohesive and dispersive OVDs compared to the AcrySof material which has a higher EWC. Thus, with the slight increase in the hydrophilicity of the Clareon material, a prolonged removal time might be expected.

This study aims to compare the removal times of a cohesive OVD after implantation of the Clareon CNA0T0 IOL to the ones after implantation of the AcrySof SN60WF IOL, using a laboratory setup with porcine eyes.

## Materials and methods

### Intraocular lens implantation

Twenty fresh porcine cadaver eyes (FVZ Mannheim GmbH, Mannheim, Germany) were prepared for the Miyake-Apple posterior view technique and assessment of OVD removal following IOL implantation^5,6^. The natural lens of each eye was removed via standardized phacoemulsification using a Megatron S3 (Geuder AG, Heidelberg, Germany) with a flow rate of 25 ml/min and vacuum of + 500 mmHg (maximum irrigation/aspiration [I/A])^1^. Two paracenteses were performed at the 3- and 9-o’clock positions, and a 2.5-mm corneal incision was created at the 12-o’clock position. A capsulorhexis of approximately 5 mm was made using an Utrata forceps. IOL implantation was only performed in eyes with a round capsulorhexis and an intact capsular bag.

To allow enhanced visualization of the OVD’s presence in the porcine eye, 0.05 mg Fluorescein (Fluorescite 10%, Alcon, Fort Worth, TX, USA) was added to 1 ml of ProVisc^®^ OVD (sodium hyaluronate 1%, Alcon, Fort Worth, TX, USA)^1,2^. After lens removal, the capsular bag and anterior chamber in each eye were filled with 1 ml of fluorescein-stained OVD. Fluorescein-stained OVD was also inserted in the Monarch III D injector cartridge (Alcon, Fort Worth, TX, USA) to lubricate IOL delivery. Ten IOLs of each model (20.0 D Clareon CNA0T0 and 20.0 D AcrySof SN60WF) were implanted in freshly enucleated porcine cadaver eyes with an intact anterior segment.

The different material properties and the specifications of intraocular devices used in this study are summarized in Table 1.

**Table 1:** Specifications of intraocular devices used in this study.

Intraocular device	Manufacturer	Material name	(Specification)	Copolymer	Equilibrium water content (in percent)
Intraocular lens	Alcon	AcrySof	(SN60WF)	PEA and PEMA cross-linked with BDDA	0.4%
Intraocular lens	Alcon	Clareon	(CNA0T0)	PEA and HEMA cross-linked with BDDA	1.5%
Ophthalmic Viscosurgical Device	Alcon	ProVisc		Sodium hyaluronate 1%	–

*PEA* phenylethyl acrylate, *PEMA* phenylethyl methacrylate, *HEMA* hydroxyethyl methacrylate, *BDDA* butanediol diacrylate.

**Figure 1:**
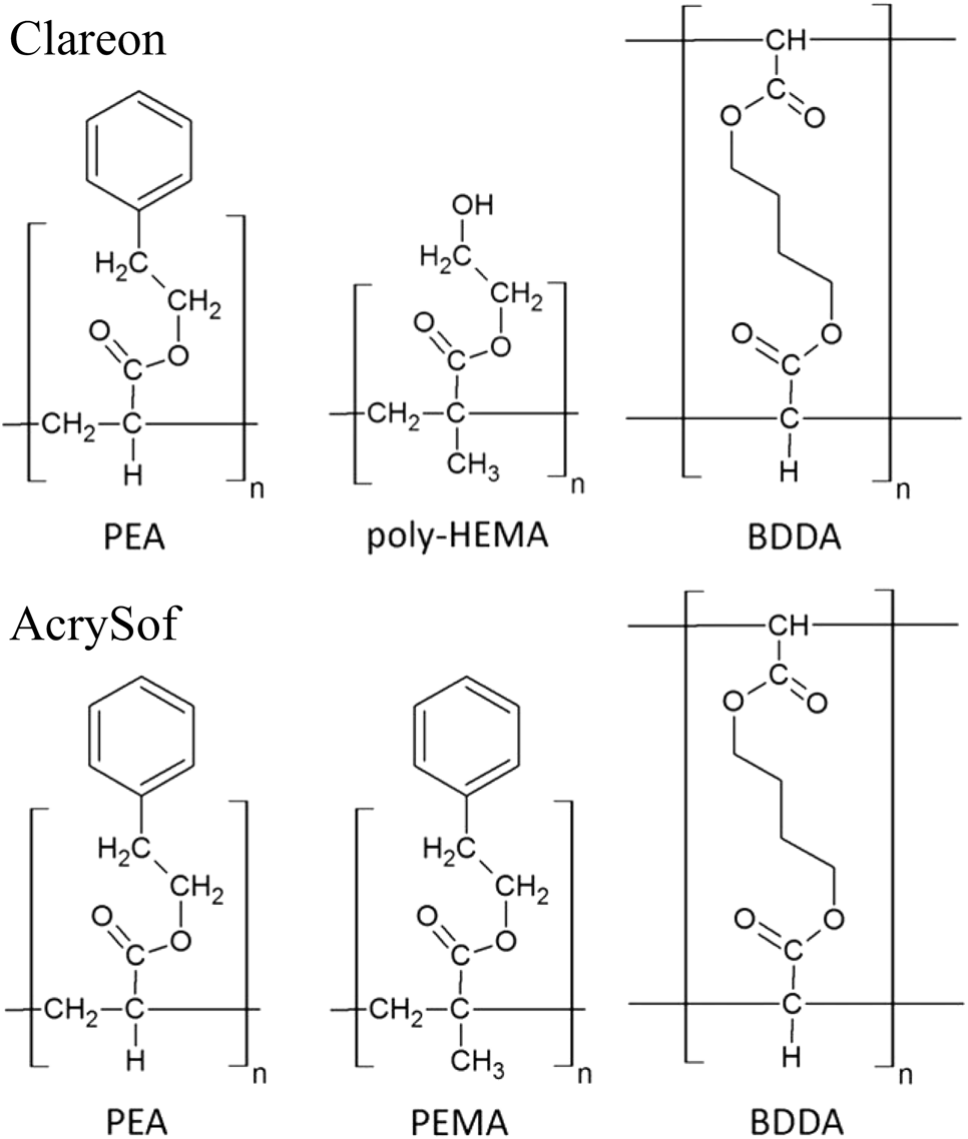
Polymer components of the IOL materials used in this study. Whereas the AcrySof material composes phenylethyl acrylate (PEA) and phenylethyl methacrylate (PEMA) cross-linked with butanediol diacrylate (BDDA), in the Clareon material the PEMA monomer is replaced with the more hygroscopic monomer, 2-hydroxyethyl methacrylate (HEMA). Figure modified from Ref.^8^.

In the Clareon material, the phenylethyl methacrylate monomer takes the place of the more hygroscopic monomer, hydroxyethyl methacrylate (Fig. 1)^7^.

### Ophthalmic viscoelastic device removal time

The stained OVD was removed immediately following IOL implantation as presented in previous studies from our laboratory by means of the “rock and roll” method as first described by Arshinoff^19^; using a flow rate of 40 ml/min and a vacuum setting of + 500 mmHg maximum, the I/A tip was “rocked” back and forth on the anterior side of the IOL to facilitate aspiration of the OVD^1,2^. Any OVD that remained on the IOL’s posterior side was removed by placing the I/A tip underneath the lens to aspirate it. All the procedures were performed by an experienced surgeon (GUA). The Miyake-Apple posterior view technique was used for posterior photography and video analysis of the stained OVD removal^5,6,9^. The I/A procedure was timed on the video counter to measure the total OVD removal time for each lens, and mean removal times for each lens type were calculated.

### Statistics

A priori sample size calculation was conducted using G*Power v. 3.1 based on the results of a previous laboratory study comparing the removal times of cohesive OVD between acrylic IOLs with different equilibrium water content: a total number of 16 eyes (8 per group) is required to achieve a 80% chance of detecting a difference at a 5% level of significance (2 tailed)^1,10^. To account for possible dropouts, two additional eyes were included for each group. Nonparametric statistical analyses was conducted using the Mann–Whitney U test (Minitab 17, State College, PA, USA).

## Results

**Figure 2:**
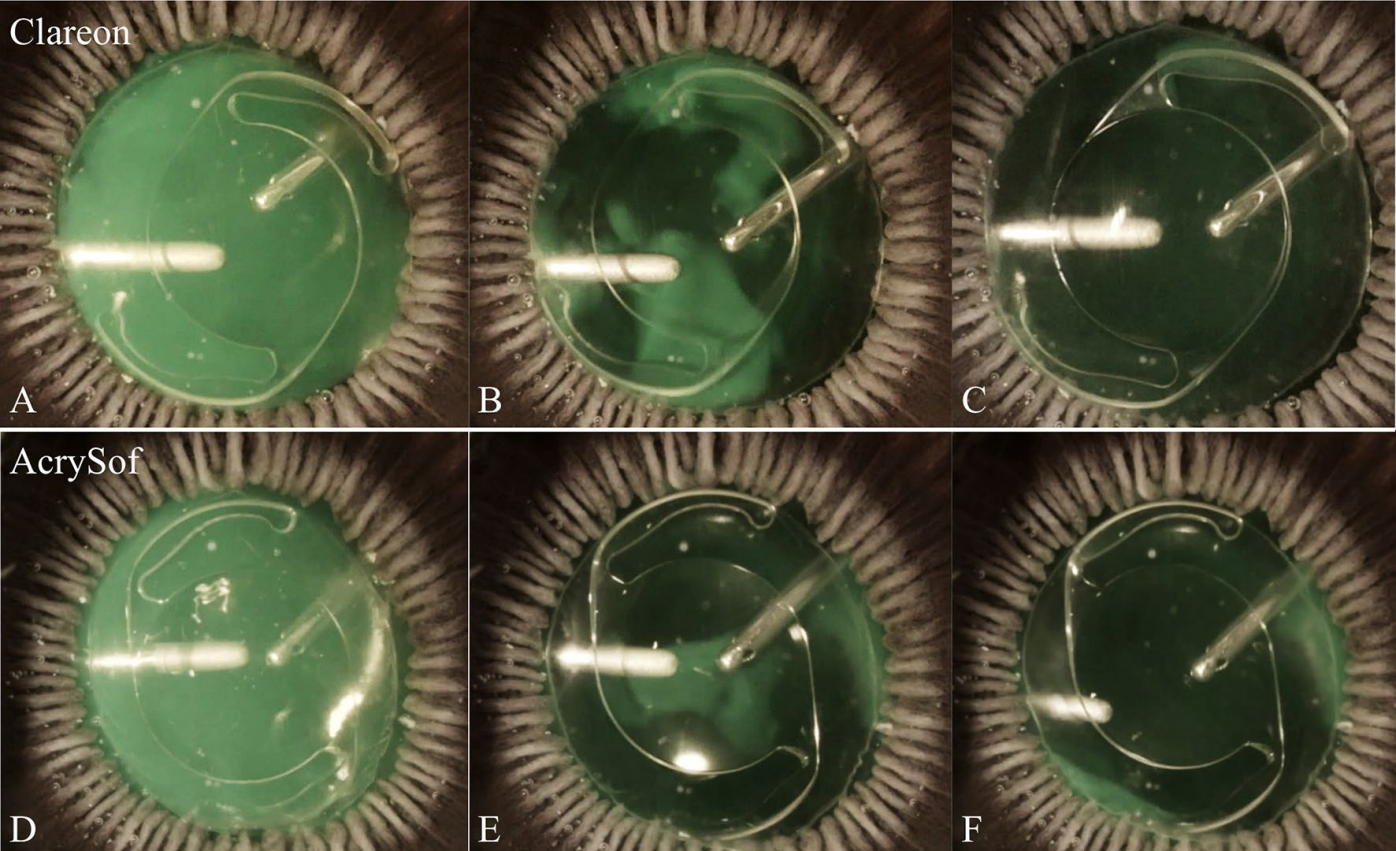
Course of the cohesive ophthalmic viscoelastic device (OVD) removal (Miyake-Apple posterior view) with a Clareon CNA0T0 IOL (**A**–**C**) and an AcrySof SN60WF IOL (**D**–**F**). Beginning of the irrigation/aspiration procedure (**A**/**D**), course of removal with some remaining OVD (**B**/**E**) and complete OVD removal (**C**/**F**).

Surgical procedures including phacoemulsification and IOL implantation of both lens types were performed successfully and OVD was removed completely from all eyes without any complication; however, one eye was excluded from each group because completion of OVD removal could not be clearly visualized due to insufficient clarity of the cornea. The Miyake-Apple posterior view technique was used in one examplary eye of each group to vizualise the OVD removal behavior during the course of irrigarion/aspiration (Fig. 2).

**Figure 3:**
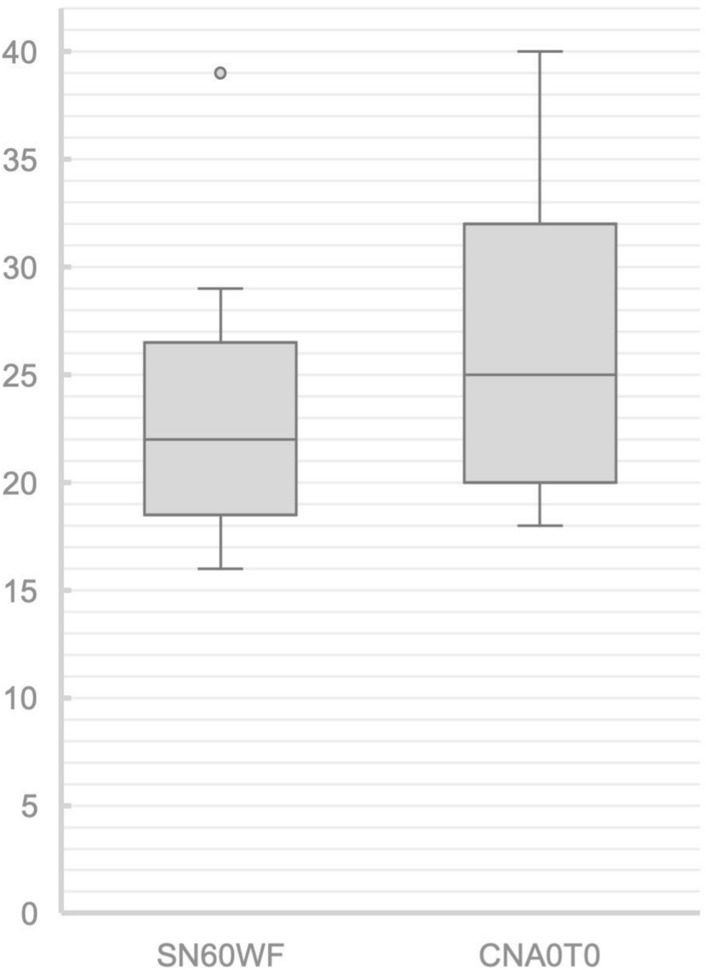
Boxplot of the comparison of removal times of the stained OVD for the Clareon CNA0T0 (n = 9) and AcrySof SN60WF (n = 9) IOLs; there was no significant difference (*P* = 0.27, Mann–Whitney).

For the Clareon CNA0T0 IOL, cohesive OVD removal time ranged from 18 to 40 s with a mean removal time of 26.4 ± 6.8 s. For the AcrySof IOL, cohesive OVD removal time ranged from 16 to 39 s with a mean removal time of 23.6 ± 6.6 s. There was no statistically significant difference in removal times between the 2 IOL models, *P* = 0.27 in Mann–Whitney test (Fig. 3).

## Discussion

The Clareon IOL is composed of an advanced hydrophobic acrylic polymer material that maintains the biomechanical attributes of the AcrySof (Table 1)^18,20^. A key property of any new lens material is its interaction with OVDs, which are essential surgical tools for cataract replacement^1,2^. During phacoemulsification, OVDs play an important role in preserving the shape of the eye by stabilizing the anterior chamber and protecting the corneal endothelium^4,11–13^. OVDs also facilitate IOL implantation by aiding lens advancement in the IOL delivery system; this helps ensure that the lens is injected into the eye without causing damage to the IOL or the surrounding tissue^12^.

Despite the protective role OVDs play during lens removal and IOL implantation, they may contribute to increased postoperative IOP in the anterior chamber if they are not entirely removed after the implantation procedure^1,2,4,14^. Increased IOP can damage the corneal endothelial cells, and in some cases may damage the optic nerve^15^. The time required for complete OVD removal may be correlated to endothelial cell loss^4^. Therefore, OVD selection and removal characteristics are important surgical considerations.

Dispersive OVDs are composed of short molecular chains that adhere to the corneal endothelium^4,16^. Although this provides protection against damage during surgery^1,16^, adherence to the endothelium makes OVD removal difficult and may require extra time. Cohesive OVDs, including ProVisc, are made up of long molecular chains and help maintain the shape of the eye during surgery. Generally, these are easier to remove after IOL implantation^1,4^. Viscoadaptive OVDs, which act either cohesively or dispersively, were developed to provide the benefits of both OVD types: protection of the eye and efficient removal^13^. However, high-viscosity viscoadaptives adhere more to hydrophobic acrylic IOLs, thus prolonging removal time. The IOL type chosen for a particular surgical case helps determine the appropriate class of OVD to use with that IOL, and both of these devices (the IOL and the OVD) will influence the removal technique^2,15^.

The physical design of the lens (such as optic size, geometry, and haptic angle) can affect OVD removal time, and so can the IOL’s material composition^1,2^. In a comparative study of lenses made from acrylic, silicone, or PMMA, lenses were implanted using cohesive or dispersive OVDs^1^. The average removal times were longer for all lens types when the dispersive OVD was used (Viscoat, 35.5 ± 10.0 s) versus the cohesive OVD (ProVisc, 25.6 ± 4.7 s)^1^. In an additional study, removal times were long for a high-viscosity cohesive OVD, Healon5^®^, (34.1 ± 1.2 s) when used with the acrylic IOL^2^.

Foldable acrylic lenses require only a small surgical incision^2^; however, OVD might be left trapped behind the IOL, between it an the posterior capsule^15^. This can make OVD removal challenging^2^, and additional time is needed to remove any OVD on the posterior side by placing the I/A tip underneath the lens as described above^2,15^.

As we used a cohesive OVD for this study, results are only pertaining to cohesive OVDs and cannot be applied to OVDs in general. In previous studies to evaluate OVD removal times, enucleated cadaver eyes were used for the assessments; however the cornea and iris were removed, making them void of an anterior chamber. As a result, OVD was only removed from the IOL and capsular bag and did not include the anterior chamber. In the current study, porcine eyes were used with an intact anterior segment, necessitating OVD removal from both the capsular bag and the anterior chamber. Furthermore, a larger volume of viscoelastic was used in this study due to the anatomical dimensions of the anterior chamber of the porcine eye. These results show that following Clareon IOL implantation, cohesive OVD removal times for ProVisc are similar to the results for AcrySof IOL implantation and also similar to the ones obtained in previous studies^17^.

## Conclusion

Both IOLs used in this study were hydrophobic acrylic lenses; the Clareon with a slightly higher water content (1.5%) compared to the AcrySof (0.4%) (Table 1). However, based on the results of this study both IOL models have similar cohesive OVD interactions and ease of removal.

There were no unexpected complications during OVD removal for either lens model. Cohesive OVD removal times of approximately 25 s for the Clareon and AcrySof IOLs demonstrated that both IOL materials allowed for similar ease of cohesive OVD removal. The Clareon CNA0T0 IOL showed no significantly different cohesive OVD removal time with ProVisc compared with the AcrySof IOL. Future work may assess the interaction of the Clareon CNA0T0 IOL with OVD in a clinical setting.

## Data Availability

All data generated or analysed during this study are included in this published article.
